# Evaluation of Internal and Marginal Shrinkage Stress in Adhesive Class III Cavities Restored with Different Resin Composite Combinations—A 3D-FEA Study

**DOI:** 10.3390/dj13080367

**Published:** 2025-08-14

**Authors:** Elisa Donaria Aboucauch Grassi, Guilherme Schmitt de Andrade, Ana Beatriz Gomes de Carvalho, Roberta Gasparro, Mauro Mariniello, Angelo Aliberti, Pietro Ausiello, Alexandre Luiz Souto Borges

**Affiliations:** 1 Department of Dental Materials and Prosthodontics, Institute of Science and Technology, São Paulo State University (UNESP), 777th Eng. Francisco José Longo Av., São José dos Campos 12245-000, SP, Brazil; elisa.aboucauch@unesp.br (E.D.A.G.); ana.b.carvalho@unesp.br (A.B.G.d.C.); alexandre.borges@unesp.br (A.L.S.B.); 2 Private Practice, Schmitt Odontologia, Cascavel 85801-031, PR, Brazil; guisdandrade@hotmail.com; 3 Department of Neuroscience, Reproductive Science and Odontostomatological Sciences, University of Naples Federico II, Via Sergio Pansini 5, 80138 Naples, Italy; roberta.gasparro@unina.it (R.G.); mauro.mariniello@gmail.com (M.M.); pietausi@unina.it (P.A.)

**Keywords:** finite element analysis, polymerization shrinkage, composite resins, class III dental restoration, dental materials, dental restoration

## Abstract

**Objectives:** To study the effects of internal and marginal polymerization shrinkage stress and distribution in different resin composite class III dental restorations in relation to the restorative technique using numerical finite element analysis (FEA). **Methods:** A 3D model of a human hemi-maxilla with a sound maxillary central incisor were created. Four class III distal cavities were shaped and differently restored. Four groups of resin composite combinations were analyzed: group C (three increments of conventional composite); group B (two increments of bulk-fill composite); group FC (flowable base + three increments of conventional composite); and group FB (flowable bulk-fill base + two increments of conventional composite). The resulting four models were exported to FEA software for static structural analysis. Polymerization shrinkage was simulated using thermal analogy, and stress distribution was analyzed using the Maximum Principal Stress criterion at the marginal and internal cavity interfaces. **Results:** Group FC showed the highest stress at the level in the proximal region (9.05 MPa), while group FB showed the lowest (4.48 MPa). FB also exhibited the highest internal dentin stress, indicating potential risks for long-term bond degradation. In the cavo-surface incisal angle, the average peak stress across all groups was 3.76 MPa. At the cervical cavo-surface angle, stress values were 3.3 MPa (C), ~3.36 MPa (B), 3.41 MPa (FC), and 3.27 MPa (FB). **Conclusions:** Restorative technique did not significantly influence marginal stress distribution in class III composite restorations. However, the bevel area at the cervical margin showed the highest concentration of shrinkage stress.

## 1. Introduction

The durability of Class III resin composite restorations is a relevant concern for patients, as these restorations are located in the anterior esthetic zone and are visible when smiling [1]. Recently introduced hybrid and nanocomposite light-curing resin materials are easier to handle and polish than earlier generations [2], favoring both the esthetics and longevity of restorations over time [3]. An important factor influencing the final esthetic outcome of Class III adhesive restorations is the presence of a 0.5 mm-wide, 45° enamel bevel, which allows the restoration margins to blend into the tooth structure [2,3]. Despite advancements in light-curing resin-based composites, polymerization shrinkage remains an unavoidable drawback during the setting process. In cavities with high configuration factors (C-factor) or where substantial amounts of enamel and dentin are missing [4], polymerization and occlusal loading can generate stresses that compromise the bonded interfaces, potentially leading to marginal leakage [5]. Leakage refers to the penetration of fluids, bacteria, and ions between the restorative material and the cavity walls, which may result in pulp irritation, tooth discoloration, and secondary caries, as factors associated with clinical failure of adhesive restorations [6].

The polymerization kinetics of methacrylate-based resins typically result in post-gel volumetric shrinkage ranging from 1% to 4.5%, depending on the degree of conversion of monomers into long-chain polymers [7,8]. To address this, new monomers have been developed to minimize shrinkage during polymerization inside the cavity [9,10,11]. The improved rheological and viscoelastic properties of these monomers contribute positively to this goal [12,13,14]. In silico studies have validated in vitro and in vivo findings, showing that flowable composites, when combined with incremental layering techniques, can reduce shrinkage stress and stress caused by occlusal loading [4,15]. Additionally, resins with a lower elastic modulus demonstrate greater capacities for deformation, further reducing shrinkage stress in dental restorations [16]. The application of flowable composites and the use of layered resin techniques have thus been proposed to mitigate polymerization shrinkage effects [17]. However, as layering techniques require more clinical time, new composite formulations have been introduced to allow bulk-filling in a single increment. Further scientific and clinical evidence is still necessary to fully validate the performance of bulk-fill composites [18,19].

Given that both the material properties and the restorative technique can influence the success of Class III restorations, the present study aimed to evaluate the effect of polymerization shrinkage on stress distribution in these cavities using Finite Element Analysis (FEA). This in silico method is a well-established mathematical tool for analyzing the mechanical behavior of restorative materials and tooth structures [15,16,20,21]. The main hypothesis is that the two variables under investigation, incremental technique and composite resin type, affect the distribution of polymerization shrinkage stress in Class III adhesive restorations. The null hypothesis is that neither variable significantly influences stress distribution.

## 2. Materials and Methods

**Figure 1:**
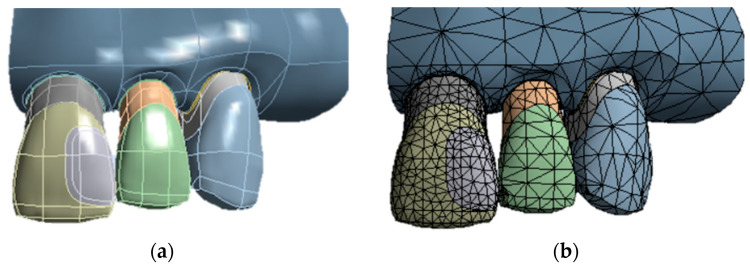
Left Hemi-maxilla modeling (**a**); Model mesh representation (**b**).

In a 3D CAD modeling software, Rhinoceros (version 7.0SR8 McNell, North America, Seattle, WA, USA), a left hemi-maxilla was modeled presenting a distal class III dental cavity in the maxillary central incisor with a cavo-surface bevel on the buccal surface (6 × 4 × 3.75 mm, with a 0.5 mm bevel on the buccal surface) (Figure 1a).

The dental class III model contains enamel, dentin, and chamber and pulp tissue and was exported to the finite element analysis software (ANSYS 2021.R1, ANSYS Inc., Houston, TX, USA) in order to analyze the polymerization shrinkage through thermal analogy. The mesh of the models was made up of triangular and tetrahedral elements (Figure 1b), including 199,640 elements and 379,070 nodes in average for the 4 model groups analyzed and was submitted to a mesh convergence test (10%), avoiding possible discrepancies in the results. The geometry was fixed on the surface of the cortical bone of the sectioned maxilla, and the shrinkage was simulated by thermal analogy in each layer separately using the “birth” and “death” element of the Ansys software (ANSYS 2021.R1, ANSYS Inc., Houston, TX, USA).

The Maximum Principal Stress was adopted to assess the effects due to the applied investigation conditions. The stress distribution at the adhesive interfaces were analyzed. The materials involved in the solid 3D models were considered isotropic, homogeneous, and linearly elastic. All materials and biological components were considered perfectly bonded to each other, and each layer of restorative material was simulated to polymerization shrinkage, following the steps of each ‘technique’. To simulate the effect of polymerization shrinkage of the composite resin-based materials and the adhesive interface, a linear thermal expansion of 0.01 was used, and the coefficient of thermal expansion used for the materials is described in Table 1 [21,22,23,24,25].

No resin bonding layer between the cavity and the first resin composite filling material was simulated because of a not significant and neglectable thickness (10 micron) [26]. The thermal analogy simulates the effect of polymerization shrinkage by attributing a one-degree drop in temperature to the adhesive interfaces of the composites with the substrate, developing a contraction of the resin and the adhesive material, and thus generating tension between the tooth and the restoration. Each of the tissues in the tooth models was defined in terms of the mechanical properties (Young’s modulus and Poisson’s ratio). The data are listed in Table 1.

**Figure 2:**
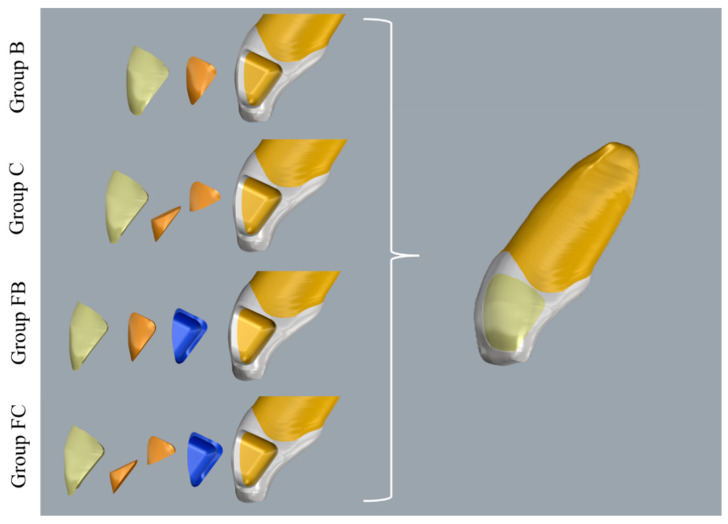
Groups division.

The groups were divided according to the restorative technique used, as follows (Figure 2): GROUP B—upper central incisor cavity with cavo-surface bevel + restorative technique using a single increment of bulk-fill resin (Bulk Fill Z350 3M™ Filtek™ 3M Company, St. Paul, MN, USA) + a single resin increment of enamel closing the cavity (Filtek Z350 XT 3M™ Filtek™ 3M Company, St. Paul, MN, USA); GROUP C—upper central incisor cavity with cavo-surface bevel + conventional restorative technique with two increments of dentin composite resin (Filtek Z350 XT) + a single resin increment of enamel closing the cavity (Filtek Z350 XT); GROUP FB—upper central incisor cavity with cavo-surface bevel + resin coating with bulk-fill fluid resin (Bulk Fill Flow Z350) + conventional restorative technique with one increment of dentin composite resin (Filtek Z350 XT) + a single resin increment of enamel closing the cavity (Filtek Z350 XT) and GROUP FC—upper central incisor cavity with cavo-surface bevel + resin coating with bulk-fill fluid resin (Bulk Fill Flow Z350) + conventional restorative technique with two increments of dentin composite resin (Filtek Z350 XT) + a single resin increment of enamel closing the cavity (Filtek Z350 XT). All the restored cavities had a final volume of 47, 35 mm^3^. This volume was differently filled in reason of the materials and layering techniques Group as described in Figure 2 and plotted in Table 2.

As the analysis was performed considering a non-failure condition, all the materials were assumed to behave as elastic materials throughout the entire deformation. Polymerization shrinkage was simulated by thermal analogy, reproduced by lowering the temperature by 1 °C, and entering the coefficient of linear thermal expansion [5,26,27].

## 3. Results

**Figure 3:**
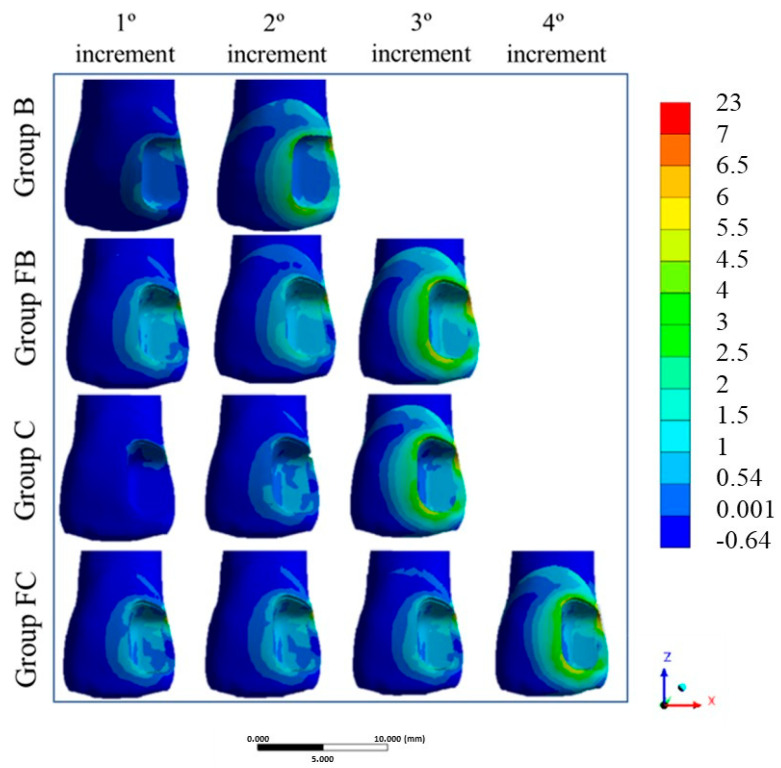
Maximal Principal Stress in each increment (MPa).

Each restorative technique was categorized based on the number of increments used, simulating the corresponding polymerization stages. A colorimetric scale was employed to illustrate stress concentrations, where warmer colors indicate higher shrinkage stress, and cooler colors represent lower stress levels. Therefore, Group B received two increments, Groups FB and C received three, and Group FC received four polymerization increments (Figure 3).

For the enamel adhesive interface, group FB, where three resin composites increments were considered, had the highest peak stress of 10.3 MPa, followed by group FC (9.2 MPa), where four increments were applied, and groups B, where two increments were considered, and C, where three increments were used, had the same peak stress of 7.6 MPa. Regarding the dentin adhesive interface, groups B and C had the lowest peak stress values, respectively (2.3 MPa and 2.9 MPa), followed by groups FC (4.1 MPa) and FB (4.2 MPa) (Table 3).

**Figure 4:**
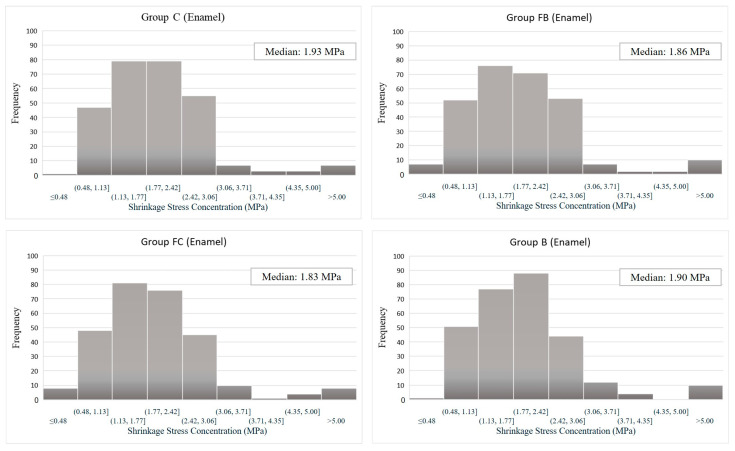
Histogram—Shrinkage Stress Concentration (Enamel) vs. Loading Frequency.

Although groups FC and FB showed higher stress concentrations than groups C and B in the enamel region, they all showed a higher frequency of stress concentration of around 1.83–1.93 MPa (Figure 4). Accordingly, the four groups showed a homogeneous distribution in the enamel region.

**Figure 5:**
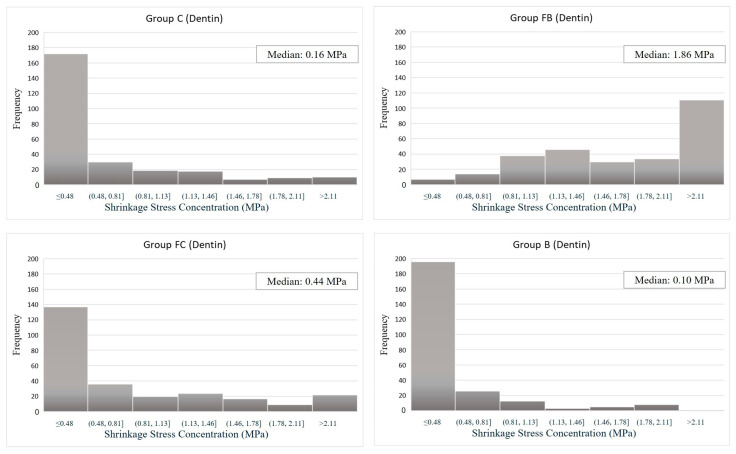
Histogram—Shrinkage Stress Concentration (Dentin) vs. Frequency.

In the dentin region, the FB group showed a stress concentration of 1.86 MPa at the highest intensity (Figure 5), followed by the FC (0.44 MPa), C (0.16 MPa), and B (0.10 MPa) groups. In this respect, groups C and B had the lowest stress concentrations at the highest frequency, with a similar distribution between them. Group FC, on the other hand, had a higher peak stress (4.1 MPa), so the stress distribution was higher under 0.44 MPa. The FB group, which had the highest stress concentration (4.2 MPa), showed a higher frequency distribution at 1.86 MPa, substantially higher than the other groups, though lower than its own peak.

## 4. Discussion

The null hypothesis, that there would be no differences in terms of shrinkage stress distribution internally and at the margins among class III cavities restored using either bulk-fill or conventional resin composites, alone or in combination with a bulk-fill flowable resin and different layering techniques, to fill the cavity volume with a final mass of 47.35 mm^3^ resin composite materials, was rejected. The results of this in silico investigation showed that the four simulated models exhibited small differences in stress magnitudes, but these were not significant enough to affect the overall behavior of the restored tooth. Although assuming linear, homogeneous, and elastic material behavior in finite element analysis (FEA) simplifies the complex biomechanics of dental tissues and restorative materials, this approach remains widely accepted for theoretical and comparative studies. Despite its limitations in capturing nonlinear or time-dependent responses, it enables standardized simulations that allow for reliable comparisons between restorative strategies under identical conditions. Thus, the present FEA model provides valuable insights into stress distribution associated with polymerization shrinkage and helps identify biomechanical trends among techniques for Class III restorations.

The bonding layer was excluded from the model due to its minimal thickness (~10 µm), which complicates meshing without significantly affecting stress distribution under static conditions. While this simplification may impact absolute values at the interface, it does not compromise the comparative evaluation across groups. Nonetheless, this limitation is acknowledged, and future studies including nonlinear material properties and adhesive layer simulation are encouraged to enhance clinical relevance.

One of the clinical consequences of the polymerization shrinkage in adhesive dental resin composite restorations is the formation of gaps and microcracks [27]. These defects can lead to marginal staining, degradation of the adhesive interface, and eventual leakage [28]. The single increment bulk-fill composite resin technique has shown promising success in clinical practice, supporting the use of bulk materials also in 4 mm deep cavities. The potential lower polymerization shrinkage stresses during setting theoretically reduce the disrupting effects at the adhesive interfaces in dental restorations [12,13,14]. Risks of microleakage and postoperative sensitivity have been consequently excluded. Other in vitro investigations, however, suggest it is still necessary to monitor these new one single increment class of resin composite materials [18]. This seems particularly true in large posterior cavities where lost enamel and dentine volumes were severe and had to be restored with large mass of resin composites [21]. The incremental technique, on the contrary, using conventional composite resins by adequate layering in dental cavities is, today, popularized and used on a large scale [17]. Conversely, many studies show the use of the incremental filling technique displays a lower polymerization stress concentration in large composite resin restorations compared with the single increment bulk fill technique [25,29,30]. Other investigations suggest taking into account the shape of the cavity, the c-factor, and the volume of the cavity to be restored before introducing the restorative technique [31] and to always use flowable composites as a liner and internal stress absorber materials [30].

Results obtained in this study carried out in adhesive class III simulated restorations showed that Group B and C, where two or three increments of different materials (with similar elastic modulus of 13.45 GPa and 13.46 GPa, of conventional or bulk-fill resin composite materials, with no flowable composite internally layered), respectively, were placed, and did not lead to a different stress distribution either in enamel (7.6 MPa) as or dentine (2.9 and 2.3 MPa), as plotted in Table 3. In Groups FB and FC, the introduction of a flowable resin composite (E = 7.95 GPa) in combination with two or three increments of conventional resin composite (E = 13.45 GPa) showed higher values of shrinkage stress in dentine and in the enamel area than Groups B and C. In this sense, the presence of a flowable, with a lower filled resin organic monomers matrix (% vol) as first layer, has induced a higher polymerization shrinkage percentage internally in dentine, as well as marginally at the enamel surfaces, delivering a very complex behavior of these materials, as already investigated by Baroudi et al. [32]. These data validate the results obtained by the review and meta-analysis study by Kunz et al. [33] in which the authors attest that the clinical performance of incremental resins compared to bulk-fill resins are similar. In this study, anyway, class II dental restorations were considered.

The bevel region in the enamel showed a higher stress concentration for all groups, which may be due to the size of the bevel, as the smaller the angle, the greater the stress concentration. This result upholds the studies by Martins LC et al. [24]. They compared class V adhesive dental restorations with different techniques and composite resins materials, and it was noted that the bevel region presented a higher stress concentration. Martins LC et al. also points out in an in silico investigation that by increasing the number of layers using a lower size of restorative material at each increment, the residual stress in the model is not reduced; therefore, the choice of technique can result in a significant difference. When we compare the technique using the bulk-fill resin vs. the conventional resin by increments, the bulk-fill resin presents lower stress concentrations with peaks of lower magnitude [24]. However, this is mainly due to the structural characteristics of the composites and to their physical properties. In the present study, there was no qualitatively significant difference between the restorations, most likely due to the techniques used being recommended for the resins applied. This is because bulk-fill resins were developed with mechanical properties that favor the use of larger increments during restoration preparation; however, the use of conventional resins with single increments and with a greater volume can impair the polymerization of the composite and cause failures in the restoration [31,34]. It is important to emphasize that, in the study conducted by Martins LC et al. [24], stress distribution was simulated in a Class V cavity of a premolar. The present investigation, however, focuses on Class III cavities in maxillary central incisors, which are more esthetically demanding and exhibit different biomechanical behavior due to their anatomical and geometric characteristics. Furthermore, this study evaluates distinct restorative protocols, including the use of a resin coating with a flowable bulk-fill composite followed by conventional resin increments, a technique that is clinically relevant but underexplored in Class III restorations.

The choice of technique is very important for the success of restorations but within the results obtained in the present study where medium size class III dental cavities (47.5 m^3^) were investigated and the use of numerous increments of resin composites seems to not support a lower shrinkage stress distribution. The high marginal stress peaks at the enamel interface in all the groups investigated can be explained considering the mismatch existing between the enamel modulus (80 GPa) and the resin composite one (13.45 GPa). In this analysis, we postulated a perfect bonding among the different materials surfaces, and an isotropic linear, elastic behavior of all the tested materials. Polymerization shrinkage stress distribution was more frequently concentrated marginally, at the enamel region, with similar patterns observed across all groups. Adhesion to enamel was in vitro and in vivo achieved through resin bonding micromechanical retention created by the dissolution of hydroxyapatite with phosphoric acid, as introduced by Buonocore in 1955, followed by the penetration of adhesive into the resulting microporosities [35,36]. This bond is more stable and long-lasting than the adhesion of bonding agents to dentin, which is more complex due to its hydrophilic nature [35,37]. Clinically, the higher frequency of stress concentration in the enamel region (cavo-surface bevel) is unlikely to compromise the quality of the restorations; in this in silico investigation, regardless of the technique employed, we assumed perfect bond between enamel and the resin composite layer.

Regarding dentin, Group FB exhibited a higher concentration of stress, which could negatively impact adhesion to this substrate, given the greater complexity of bonding to dentin. Group FB exhibited the highest stress concentration in dentin (4.2 MPa), likely due to the combination of a low-modulus flowable bulk-fill base (7.95 GPa) and only one increment of conventional composite above it. This setup may have generated a more significant volumetric shrinkage mismatch, and a less effective stress dissipation compared with Group FC, where additional layering allowed for better control of the shrinkage vectors. The modulus mismatch between flowable and conventional composites, along with the limited number of increments, may have amplified stress concentration in the dentin substrate, where bonding is more susceptible to disruption due to its organic and moist nature. Polymerization shrinkage is influenced by multiple variables, especially the resin type and configuration factor (c-factor) [38,39].

To address this, the restoration technique using small oblique increments was employed in Groups C and FC to reduce the c-factor [40], although Group FC also included dentin sealing with flowable resin. Group FC presented the second-highest median stress concentration.

The lower elastic modulus of the bulk-fill flowable resin, used for dentin filling in Groups FC and FB, may have contributed to greater internal stress transmission in the dentin region [41]. This technique, combined with the use of a single increment of conventional resin for dentin in Group FB, may have contributed to the higher stress concentration observed in dentin. Group FC, which combined dentin filling with the incremental technique (two dentin increments and one enamel increment), showed a lower stress concentration compared with Group FB, but still presented a higher stress level than Groups C and B. These data confirm the good evolution of this class of polymeric resin composite restorative dental materials in the time [42].

This study is based on a static finite element analysis (FEA) and does not simulate clinical loading conditions, such as mastication or thermal cycling. All materials were assumed to behave isotropically, homogeneously, and linearly elastically, which does not reflect the viscoelastic or time-dependent nature of dental materials. Additionally, the bonding layer was excluded from the model due to meshing limitations, which could affect stress concentrations at the adhesive interfaces. Therefore, although useful for comparative purposes, these findings should be interpreted with caution and confirmed by dynamic FEA models and long-term in vitro or in vivo studies.

## 5. Conclusions

Within the limitations of this static FEA model:

- The FB group (flowable bulk-fill base + two conventional increments) showed the highest internal dentin stress, suggesting a potentially higher risk of adhesive degradation in clinical conditions.
- The C and B groups, which avoided flowables and used either conventional or bulk-fill resins with limited increments, showed the lowest stress concentrations in both dentin and enamel.
- The use of a low-modulus flowable base, as in FB and FC, was associated with greater internal stress, likely due to elastic mismatch and shrinkage behavior.
- All techniques showed high-frequency stress concentration at the enamel cavo-surface bevel, mainly due to the modulus difference between enamel and resin composite.

These results underscore that both material selection and layering technique significantly influence stress behavior in Class III composite restorations. However, clinical decision-making should consider additional factors—including cavity morphology, esthetic demands, and operative time. Long-term in vitro and in vivo studies are essential to validate these in silico findings and refine clinical protocols.

## Figures and Tables

**Table 1:** Mechanical properties of materials.

Material	Elastic Modulus (GPa)	Poisson’s Ratio	Thermal Expansion Coefficients	Reference
Enamel	84.1	0.30	---	[20]
Dentin	18.6	0.31	---	[20]
Pulp	0.002	0.45	---	[22]
Ligament	0.069	0.45	---	[23]
Cortical Bone	13.7	0.3	---	[22]
Medullary Bone	1.4	0.3	---	[22]
Filtek Z350 XT	13.45	0.17	0.00033	[24]
Bulk Fill Z350	13.46	0.18	0.00025	[24]
Bulk Fill Flow Z350	7.98	0.32	0.00047	[25]

**Table 2:** Restored cavity partial and total Volumes (mm^3^) according to the group, to number of layers and composite resins chosen.

	Group	Group	Group	Group
Layer	B	C	FB	FC
**1**	**28.68 mm^3^** Bulk Fill Z350 (E = 13.46 GPa)	**13.77 mm^3^** Filtek Z350 (E = 13.45 GPa)	**13.21 mm^3^** Bulk Fill Flow Z350 (E = 7.95 GPa)	**13.21 mm^3^** Bulk Fill Flow Z350 (E = 7.95 GPa)
**2**	**18.67 mm^3^** Filtek Z350xt (E = 13.45 GPa)	**14.91 mm^3^** Filtek Z350 (E = 13.45 GPa)	**15.47 mm^3^** Filtek Z350 (E = 13.45 GPa)	**8.36 mm^3^** Filtek Z350 (E = 13.45 GPa)
**3**	----	**18.67 mm^3^** Filtek Z350 (E = 13.45 GPa)	**18.67 mm^3^** Filtek Z350 (E = 13.45 GPa)	**7.11 mm^3^** Filtek Z350 (E = 13.45 GPa)
**4**	----	----	----	**18.67 mm^3^** Filtek Z350 (E = 13.45 GPa)
**Total**	**47.35 mm^3^**	**47.35 mm^3^**	**47.35 mm^3^**	**47.35 mm^3^**

**Table 3:** Stress peaks for enamel and dentin at the adhesive interface in Megapascal (MPa).

**Group**	**Enamel**	**Dentin**
C (2 INC Filtek Z350 XT)	7.6 MPa	2.9 MPa
B (1 INC Bulk Fill Z350 + 2 INC Filtek Z350 XT)	7.6 MPa	2.3 MPa
FC (1INC Bulk Fill Flow Z350 + 3 INC Filtek Z350 XT)	9.2 MPa	4.1 MPa
FB (1INC Bulk Fill Flow Z350 + 2 INC Filtek Z350 XT)	10.3 MPa	4.2 MPa

## Data Availability

The original contributions presented in this study are included in the article. Further inquiries can be directed at the corresponding author.
